# Spatio-Temporal Modeling for Forecasting High-Risk Freshwater Cyanobacterial Harmful Algal Blooms in Florida

**Published:** 2020-11-02

**Authors:** Mark H. Myer, Erin Urquhart, Blake A. Schaeffer, John M. Johnston

**Affiliations:** 1 US Environmental Protection Agency, Oak Ridge Institute for Science and Education (ORISE), Athens, GA, United States; 2 US Environmental Protection Agency, Oak Ridge Institute for Science and Education (ORISE), Research Triangle Park, NC, United States; 3 US Environmental Protection Agency, Center for Exposure Measurement and Modeling, Research Triangle Park, NC, United States; 4 US Environmental Protection Agency, Center for Exposure Measurement and Modeling, Athens, GA, United States

**Keywords:** harmful algal blooms, cyanobacteria, hierarchical Bayes, integrated nested Laplace approximation, remote sensing, predictive modeling

## Abstract

Due to the occurrence of more frequent and widespread toxic cyanobacteria events, the ability to predict freshwater cyanobacteria harmful algal blooms (cyanoHAB) is of critical importance for the management of drinking and recreational waters. Lake system specific geographic variation of cyanoHABs has been reported, but regional and state level variation is infrequently examined. A spatio-temporal modeling approach can be applied, via the computationally efficient Integrated Nested Laplace Approximation (INLA), to high-risk cyanoHAB exceedance rates to explore spatio-temporal variations across statewide geographic scales. We explore the potential for using satellite-derived data and environmental determinants to develop a short-term forecasting tool for cyanobacteria presence at varying space-time domains for the state of Florida. Weekly cyanobacteria abundance data were obtained using Sentinel-3 Ocean Land Color Imagery (OLCI), for a period of May 2016–June 2019. Time and space varying covariates include surface water temperature, ambient temperature, precipitation, and lake geomorphology. The hierarchical Bayesian spatio-temporal modeling approach in R-INLA represents a potential forecasting tool useful for water managers and associated public health applications for predicting near future high-risk cyanoHAB occurrence given the spatio-temporal characteristics of these events in the recent past. This method is robust to missing data and unbalanced sampling between waterbodies, both common issues in water quality datasets.

## INTRODUCTION

Harmful algal blooms are environmental events that occur when algal populations achieve sufficiently high density resulting in possible adverse ecological and public health effects (Smayda, 1997). Harmful cyanobacteria blooms (cyanoHABs) are made up of naturally occurring photosynthetic prokaryotes found in various aquatic systems and can produce toxins (cyanotoxins). Toxic cyanoHABs are common components of Florida’s surface waters and have been identified since the late 1980s (Carmichael, 1992; Chapman and Schelske, 1997; Burns, 2008) in Florida’s freshwater and brackish environments, including those used for recreation, source waters used for drinking water supply, and finished drinking waters (Williams et al., 2001, 2007; Florida Fish Wildlife Conservation Commission, 2017). Florida freshwater cyanoHABs have consisted primarily of the genera *Microcystis, Anabaena,* and *Cylindrospermopsis* and their associated toxins: microcystins, anatoxin-a, and cylindrospermopsin, respectively (Burns et al., 2002; Phlips et al., 2002). Upon numerous sampling occasions, microcystin levels in Florida’s recreational waters have exceeded the existing World Health Organization (WHO) (Chorus and Bartram, 1999; World Health Organization, 2003) and EPA (US EPA, 2019) guidelines for cyanobacterial toxins, underscoring the potential impact of cyanoHABs on public health (Williams et al., 2007).

Several of Florida’s largest aquatic systems including Lake Okeechobee (Havens et al., 1998; Havens and Steinman, 2015); the Harris Chain of Lakes (Williams et al., 2001, 2007); and the St. Johns, St. Lucie and Caloosahatchee (Glibert et al., 2006; Boyer and FitzPatrick, 2016; United States Army Corps of Engineers, 2016) rivers have experienced the increasing adverse impacts of cyanoHABs. In 2005, the St. Johns County Department of Health released Florida’s first official health alert for a toxigenic harmful algal bloom. In response, the St. Johns River Water Management District began routine daily sampling and issued weekly releases informing the public of high microcystin concentrations and risk (Williams et al., 2007). In July 2016, concentrations (4.5 mg L^−1^) of microcystin were detected in the river-dominated Caloosahatchee and St. Lucie estuaries following very heavy rainstorms in Florida (Oehrle et al., 2017). Strong storms resulted in reservoir operators increasing the outflow from Lake Okeechobee causing the incursion of a toxic *M. aeruginosa* bloom into the St. Lucie Estuary (Boyer and FitzPatrick, 2016; United States Army Corps of Engineers, 2016).

Identification and quantification of the environmental factors that contribute to the proliferation of cyanoHABs in freshwater systems continues to be a topic of scientific research. It is generally understood that dense concentrations of cyanoHABs result from a combination of excess total anthropogenic nutrient loads, particularly phosphorus (Michalak et al., 2013). Other factors that can be important drivers of cyanoHAB abundance are positive associations with lake depth, water column stability, and water temperature (Paerl and Huisman, 2008; Taranu et al., 2012; Beaulieu et al., 2013) and a negative association with wind speed (Millie et al., 2014). Landscape alterations such as urbanization or agricultural practice can change sediment loading and further alter nutrient availability in watersheds (Lunetta et al., 2015).

Modeling efforts to identify and predict harmful algal blooms have used several approaches, including classical multivariate analysis with LOWESS smoothing (Downing et al., 2001), continuous artificial neural networks (Millie et al., 2014), linear, non-linear, and mixed-effect models (Beaulieu et al., 2014), and Bayesian modeling (Obenour et al., 2014).

These data-driven modeling efforts do not address spatial and temporal correlation in bloom occurrence. Failure to account for spatial and temporal autocorrelation violates the assumption of independent and identically distributed data and may lead to biased model estimates. Further, incorporating Bayesian inference into predictive models allows inclusion of prior knowledge, either through literature review and expert opinion or by using mathematical techniques to generate informative priors. We therefore aim to improve upon cyanoHAB models by addressing spatio-temporal correlations using Bayesian hierarchical models, leading to estimates of explanatory variable effects that are more reliable for scientific inference.

The integrated nested Laplace approximations (INLA) method (Rue et al., 2009) offers a simple way to compute complicated hierarchical models that include spatial and temporal structure. Large computational times remain a major drawback of modeling spatial correlation. However, a recent solution has been developed using stochastic partial differential equations (SPDE) to provide a faster and computationally cheaper solution to these modeling problems (Lindgren et al., 2011). R-INLA is a modeling framework developed and implemented for the statistical software R (R Core Team, 2015) that allows complex Bayesian spatial modeling with far fewer computational resources than previous approaches (Rue et al., 2017). One benefit of R-INLA is that it is applicable at any spatial and/or temporal scale. The SPDE approach allows the user to model spatial correlation and constructs flexible fields that are better able to handle complex spatial structures than alternative spatial correlation models such as kriging (Cameletti et al., 2013), making this approach appropriate for inland waterbody modeling because we see both regional clustering of blooms and vast areas of water and land with no cyanobacteria observations. See Supplemental Material 2, Statistical Appendix for definitions and discussion of these statistical terms.

In this study, we apply Bayesian spatio-temporal models using R-INLA for the purposes of mapping and predicting exceedance probabilities of World Health Organization (WHO) high-risk for recreational exposure blooms (>100,000 cells/mL) in lakes across Florida. Mapping state level cyanoHAB exceedance probabilities identifies areas where estimates are higher or lower than the state average and provides insight through visualization of patterns in both space and time in addition to inferences drawn from the effects of predictor variables.

## METHODS

### Study Area

**FIGURE 1 |:**
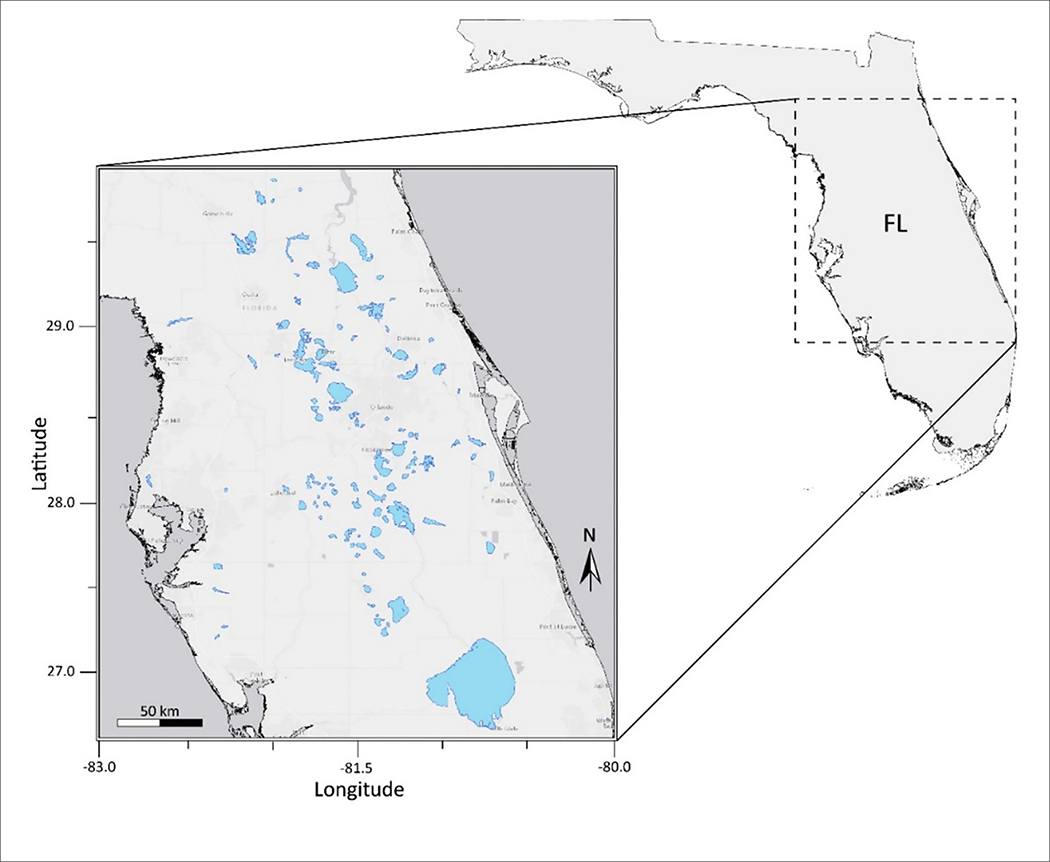
Florida study area containing inland lakes and reservoirs.

This study was conducted for 103 lakes in the state of Florida, United States. Lakes and waterbodies within three Water Management Districts: St. Johns River, Southwest Florida, and South Florida, were included in the analysis (Figure 1). Lake water pixels were extracted using the USGS/EPA Hydrography Dataset Plus (NHDPlus) version 2 (http://www.horizon-systems.com/nhdplus/NHDplusV2_home.php). All NHDPlus features classified as lakes and reservoirs were selected using U.S. Environmental Protection Agency’s 2012 National Lakes Assessment (NLA) site evaluation guidelines. Lakes in the NHDPlus shapefile with a minimum of three 300 m water pixels remaining after a land adjacency QA flag was applied were considered resolvable waterbodies. The number of pixels included in each water body ranged from 3 to 14,499. The mean and median number of pixels in each water body were 228 and 23, respectively. The number of pixels in each water body considered in the analysis are provided in Supplemental Material 2. Waterbodies classified as intermittent, estuarine, rivers, streams, or waterbodies with a surface area <27 hectares are considered “unresolvable water” and thus QA flagged based on NLA criteria. Waterbodies with missing data were excluded from statistical analysis. The OLCI satellite scene extent for our study area is located within the NLA Coastal Plain (CPL) ecoregion. According to findings from the 2012 NLA report, 23% of lakes within the CPL ecoregion pose a high risk to exposure of cyanobacteria and cyanotoxins (US EPA, 2009).

### Satellite-Derived Cyanobacteria Data Acquisition and Preparation

Satellite-derived cyanobacteria abundance data was obtained from Sentinel-3 imagery from May 2016 through May 2019 *(n =* 217 weeks). Standard OLCI Level-1B data were obtained from the NASA Ocean Biology Processing Group (https://oceandata.sci.gsfc.nasa.gov). Sentinel-3 OLCI Florida granules were in the time window of 15:20–16:00 with a frame along track coordinate of 2,520. The cyanobacteria index (CI) was calculated using a spectral shape curvature method, as originally described in Wynne et al. (2008), updated in Lunetta et al. (2015), and algorithm progression fully detailed in Coffer et al. (2020). The CI-Multi value is then converted to cyanobacteria abundance (cells/mL) as described in Wynne et al. (2010). This algorithm was previously validated across Florida watersheds (Lunetta et al., 2015; Tomlinson et al., 2016). Composite images of the maximum cyanobacteria abundance at each resolvable satellite pixel were obtained from the individual scenes within sequential 7-day (1 week) periods. Weekly maximum cyanobacteria abundance was used to minimize the effect of clouds or wind that might otherwise reduce detection of the bloom. Many cyanoHAB genera such as *Microcystis, Aphanizomenon,* and *Dolichospermum* have buoyancy control and will typically float to the surface in the day in the absence of strong winds (Visser et al., 2015). Wind can mix the blooms into the water column, diluting the surface concentration seen by the satellite (Wynne et al., 2010). Mishra et al. (2005) found satellite measures in the red region, similar to the CI-multi, typically only penetrate to a depth of 2 m or less. The OLCI sensor was selected because of public availability of data, spectral range to support deriving cyanobacteria concentrations, and 2–3 day repeat cycle (Urquhart and Schaeffer, 2020). For each water body, the satellite-derived cyanobacterial counts were averaged spatially each week to obtain one summary observation. This 1-week averaging and spatial aggregation was necessary due to cloud cover and satellite repeat cycles, though it results in a loss of information relative to using all available cyanobacterial abundance measurements without averaging and may cause short-term blooms to be missed.

### Covariates

We limited covariates that were relevant to cyanobacterial growth and were readily available at the state and U.S. national scale on a weekly basis for operational forecasting. Land use and nutrients are not readily available on a weekly time frame for the continental US. In addition, nutrient transport and availability is complex and system specific, with data lacking for most systems. Wind speed and direction were not included as it was not available for the entire OLCI time period during our analysis.

We retrieved and processed raster and GIS datasets into a set of covariates for the state of Florida at 300 m × 300 m resolution. These data were identified in our review of the cyanobacterial modeling literature as environmental determinants likely to be associated with cyanobacteria bloom occurrence including ambient temperature, surface water temperature, precipitation, and static geomorphic lake conditions (Table 1). All data were obtained from public sources. We scaled all covariates to the same resolution as our satellite imagery in order to exclude measures along the land and water interface, because we masked our lake imagery with a 300 m buffer to exclude mixed pixel contamination. Landsat surface water temperature (WTEMP) was upscaled from 30 to 300 m, while PRISM precipitation (PRECIP) and ambient air temperature (ATEMP) were downscaled from 4 km to 300 m. Upscaling is a process that transfers information from local scale to large scale. Downscaling, conversely, transfers from large scale to local scale. Processes that are heterogenous at small scales become homogenous at larger scales. All covariate data were temporally binned into weekly means by taking the arithmetic mean of 7 days within the numerical week (1 through 53) that bound each observation. Due to cloud cover and non-uniform scene acquisition, the monthly climatology of the Landsat Analysis Ready Data (ARD) surface water temperature product (Cook et al., 2014; Schaeffer et al., 2018) was used when the corresponding weekly data were not available. Spatial geographic coordinates were represented in kilometers using the Albers equal-area conic projection. Parameter-elevation Regressions on Independent Slopes Model (PRISM) mean, maximum, and minimum air temperatures and precipitation for North America was downloaded from the Oregon State PRISM Climate Group (PRISM Climate Group, 2004) through June 2019. Landsat ARD surface temperature was downloaded through USGS Earth Explorer through May 2019 in horizontal tiles 25, 26, and 27 and vertical tiles 16, 17, and 18.

All variables were averaged over all resolvable pixels within each water body each week, resulting in one summary observation per week for each water body. To aid in interpretation of relative variable influences on the response, predictor variables were centered and scaled by subtracting the mean and dividing by the standard deviation, transforming them into Z-scores. On this scale, a value of zero represents the mean, the units are standard deviations from the mean, and the value of the variable’s coefficient in the model represents the effect in log-odds of a one standard deviation increase.

To identify the set of predictor variables most appropriate for modeling inland cyanobacterial bloom presence in Florida, we used non-spatial generalized linear models (Supplementary Formula 4), implemented in the R *glmulti* package (Calcagno and de Mazancourt, 2010), confirming the choice using stepwise-selection implemented in the R *stepAIC* package (Ripley, 2002). In this way, we constructed all possible non-redundant models with every combination of covariates. The best model was determined using Akaike’s information criterion (AIC, Supplementary Formula 6), which is an estimator of out-of-sample prediction error and is used for model comparison (Akaike, 1998). See Supplementary Material for a discussion of the use of AIC. This step eliminated two variables, Maximum Lake Depth and Estimated Lake Volume. The best model, with the resulting fixed effect variables, was then used in the Bayesian spatio-temporal model.

### Hierarchical Bayesian Model Specification and Accuracy

We employed a hierarchical Bayesian spatio-temporal model to estimate exceedance probability over the WHO high-risk threshold with respect to environmental predictors. The response variable was the presence or absence of a high-risk cyanobacteria bloom, defined as a waterbody-wide average cell count above 100,000 cells/mL. Bayesian parameter estimates and prediction in the form of marginal posterior probability distributions were obtained via the R-INLA approach. For this study, weakly informative penalized-complexity priors were generated for all regression coefficients (fixed-effect parameters) and hyperparameters, allowing our large number of observations *(n =* 11,096) to inform the posterior distributions (Simpson et al., 2017). For the temporal component, we used a first order temporal autoregressive process (AR1, Supplementary Formula 5), which models the effect of time on bloom probability in each location as a function of the concentration in the previous week at that location plus an error term (Potzelberger, 1990). Spatial covariance was addressed using the INLA SPDE approach (Lindgren et al., 2011). The spatial effect represents residual error that can be attributed to location and may reflect the influence of an unmeasured or unmeasurable predictor that varies in space. We used a binomial logistic spatiotemporal model to predict harmful algal blooms (Formula 1).


Formula 1. $$logit\;\left(y_{st}\right)=\beta_{1}X_{1}+\ldots +\beta_{n}X_{n}+u_{s}+\mu_{t}$$

Generalized model structure for a binomial logistic model with spatial and temporal components.

*y*_*st*_ is the odds in favor of harmful bloom exceedance at location *s* and week *t*, *β*_1_ *…, β*_*n*_ are the *n* regression coefficients, *X*_1_*, …, X*_*n*_ are the *n* fixed independent variables, *u*_*s*_ is the value of the spatial random effect at location *s*, and *μ*_*t*_ is the value of the AR1 temporal random effect at time *t*. The *logit* is the logarithm of the odds in favor of an event and is also referred to as *log odds.* The *β*_*n*_ coefficients are in the logit or log-odds scale, which can be converted to probabilities as shown in Formula (2).


Formula 2. $$P\;(event)=\frac{e^{log-odds}}{1+e^{log-odds}}$$

Converting log-odds to probability.

The odds were converted to probabilities of harmful algal bloom exceedance for model evaluation and predictive performance evaluation. A comprehensive mathematical overview of INLA SPDE can be found elsewhere (Rue et al., 2009; Lindgren and Rue, 2015). More detailed technical explanations of INLA SPDE applied to ecological and epidemiological modeling are available (Cosandey-Godin et al., 2014; Khana et al., 2018; Myer and Johnston, 2018). See Supplementary Material for a general discussion and explanation of the statistical methods used in this study along with definitions and further equations.

The utility of the spatial and temporal effects was assessed by fitting the model with and without random effects. The models were compared using the Deviance Information Criterion (DIC, Supplementary Formula 7) to determine if the inclusion of the spatial and temporal effects improved model fit. Model performance was evaluated through holdout cross-validation in which the dataset was divided into three compartments: 80% of the data was randomly selected for training, 20% of the data was held out for validation, and the most recent week of data available at the time of the study was obtained for prediction. The model was created based on the training data only, and then model predictive power was assessed on the validation and prediction datasets. Model predictive power was determined by calculating the Area Under Curve (AUC), and by evaluating the sensitivity (true positive rate, Supplementary Formula 8), and specificity (true negative rate, Supplementary Formula 9) in predicting the holdout validation and prediction datasets after optimizing the logistic cutoff. Cutoff optimization, which chooses a value for the logistic predictor above which we consider a bloom prediction as positive, was performed by maximizing Youden’s index on the validation dataset. Youden’s index, sometimes referred to as the J-statistic, is defined in Formula (3).


Formula 3. J=sensitivity+specificity−1

Youden’s Index, or the J-statistic. Maximizing the value of this statistic provides the maximum overall accuracy for a binomial predictor.

Youden optimization attempts to find the cutoff at which sensitivity and specificity are balanced and at a maximum. All statistical analyses were conducted using R 3.3.4 on a compute cluster with 128 nodes and 4,096 cores.

## RESULTS

**FIGURE 2 |:**
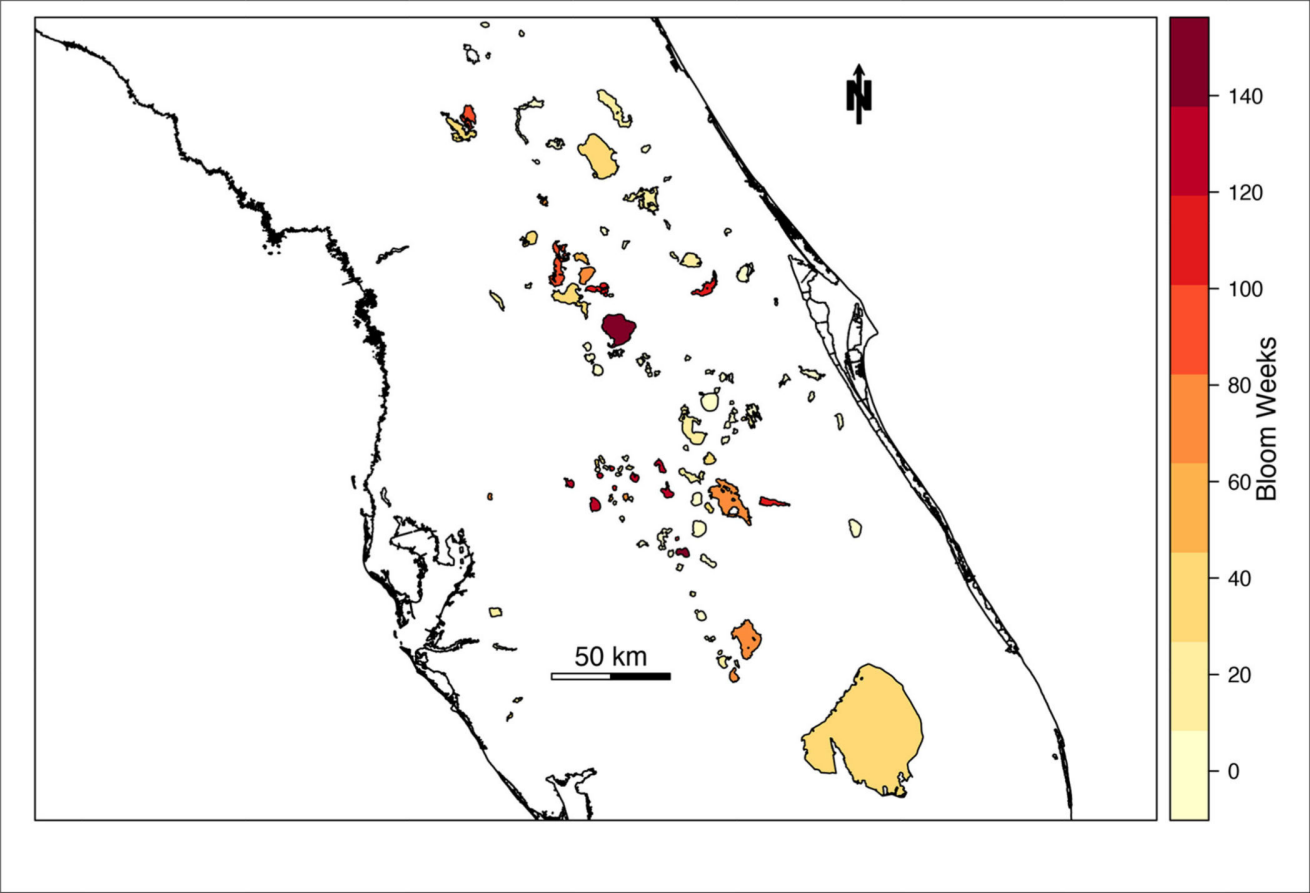
Choropleth map of the number of high-risk bloom weeks in Florida lakes from 2016 to 2019.

The likelihood of high-risk cyanoHAB presence was estimated for 103 lakes and waterbodies in Florida. The number of high-risk blooms in individual lakes across Florida ranged from 0 to 146 weeks from 2016 to 2019 (Figure 2). During the study period, 22.5% of total bloom weeks were classified as high risk (*n* = 3,149 bloom weeks). Average waterbody-wide cyanobacterial concentration in lakes classified as high-risk was 376,504 cells/mL, more than three times the WHO “high” threshold of 100,000 cells/mL.

### Model Selection

The model fit of our four candidate models was evaluated using the Deviance Information Criterion (DIC), with lower DIC values indicating better fit (Table 2). All four models included the same fixed effect predictors. The first model (M1), a nonhierarchical baseline model that did not incorporate random effects nor correlation features, exhibited a DIC of 14,195 and a ~7 s computation time. The addition of a temporal effect (M2) improved the DIC value and only slightly increased the computation time. The addition of a spatial component (M3) dramatically improved the DIC value, but approximately quadrupled computation time. The addition of the spatial and temporal autocorrelation structure (M4) further improved the DIC value but increased the computation time to ~53 s. We concluded that computational time was not an issue of concern with this model, due to the favorable computational characteristics of the INLA SPDE method. Thus, the M4 hierarchical model with a full set of time varying, fixed effect covariates and spatio-temporal correlation effects provided the best model fit and was selected as the best model with subsequent results presented as follows.

### Fixed Effect Covariates

The mean posterior coefficients of the fixed effect covariates are presented in log-odds and signify the estimated response to a one standard deviation change in the predictor variable when all other variables are held constant. For the purposes of determining statistical importance, we utilized an alpha level of 0.05 and considered a variable with a 95% credible interval that did not encompass zero to have an important effect on the response. Using stepwise-selection and the resulting AIC values, ambient air temperature, surface water temperature, precipitation, lake area, and mean lake depth were selected as fixed effect predictors in the bloom estimation models (Supplementary Table 1). Fixed effect coefficients and 95% Bayesian credible intervals for the covariates included in the full spatio-temporal model (M4) are provided in Table 3. Posterior distributions of these fixed effect variables are presented as log-odds of the scaled covariate variables. As expected, a significant positive association is observed between surface water temperature (WTEMP) and high-risk bloom presence in Florida. For a one standard deviation increase in surface water temperature (6.23°C), the expected change in high-risk bloom log odds is 0.17 (or 1.18 times greater odds). Further, mean lake depth (DMEAN) exhibited a significant positive association with high-risk bloom presence, with an increase in one standard deviation (0.75 m) leading to an expected increase in log odds of 2.70 (or 14.88 times greater odds). Ambient air temperature (ATEMP) had a significant and negative effect on the likelihood of high-risk bloom presence in Florida, with a one standard deviation increase in ambient air temperature (4.79°C) resulting in change in high-risk bloom log odds of −0.23 (or 0.79 times lower odds).

### Random Effects and Hyperparameters

**FIGURE 3 |:**
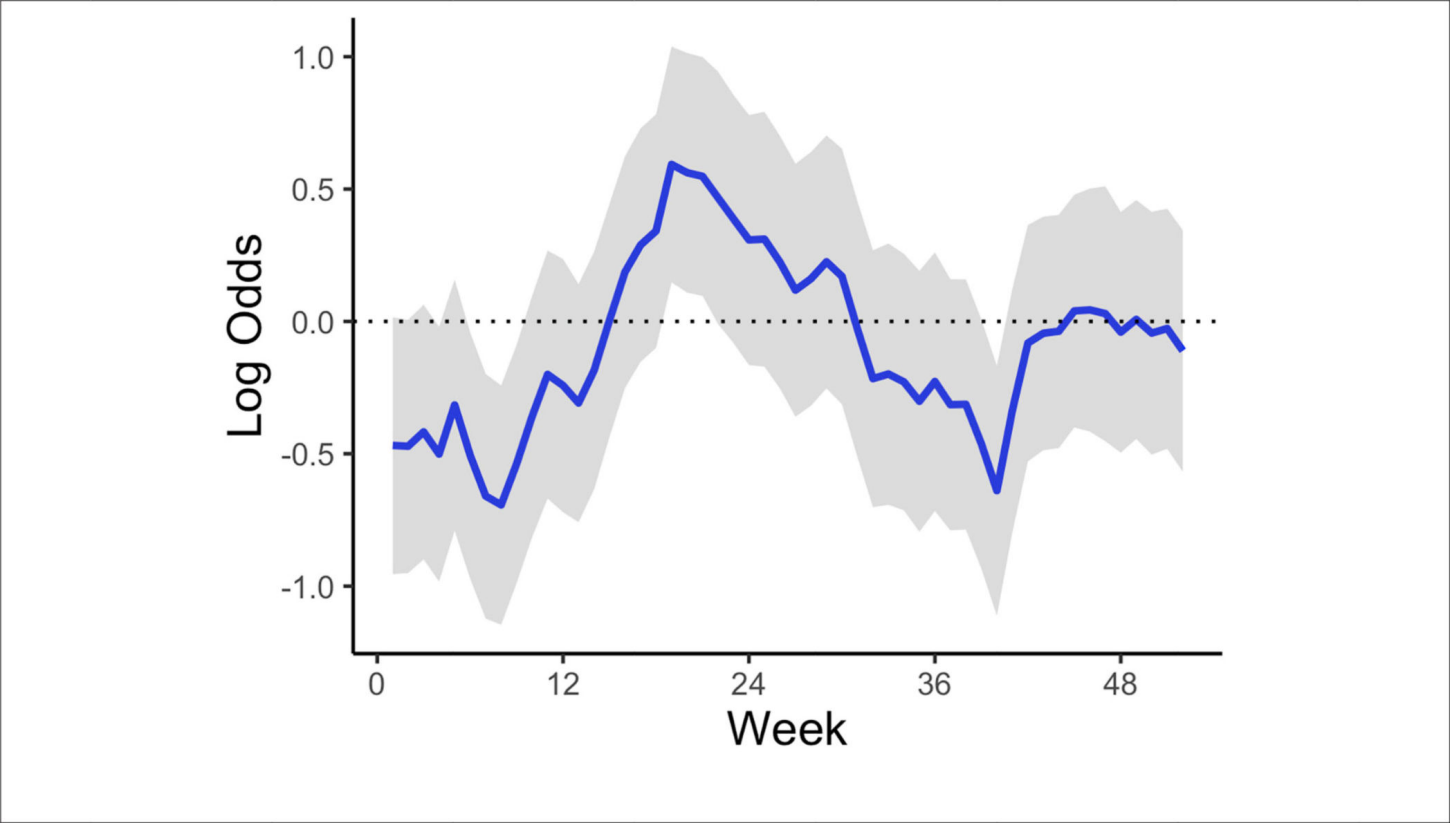
Mean AR (1) temporal trend corresponding to week of year across all study years and lakes. The shaded area represents the 95% credible interval.

The temporal model component (Figure 3) shows how the temporal effect on the odds of a high-risk bloom event vary throughout the year. This effect represents residual error that can be attributed to time and can be interpreted as the influence in the model of an unmeasured or unmeasurable predictor that varies in time. The log odds of high-risk bloom presence were higher from spring (week 15, approximately April 6) to late summer (week 35, approximately August 17) all other variables held constant. The posterior mean of the AR (1) parameter indicated an autocorrelation effect of 0.90 (95% CI = 0.68:0.99), which indicates that the temporal effect depends strongly on previous values and does not change quickly throughout the year (Table 4).

**FIGURE 4 |:**
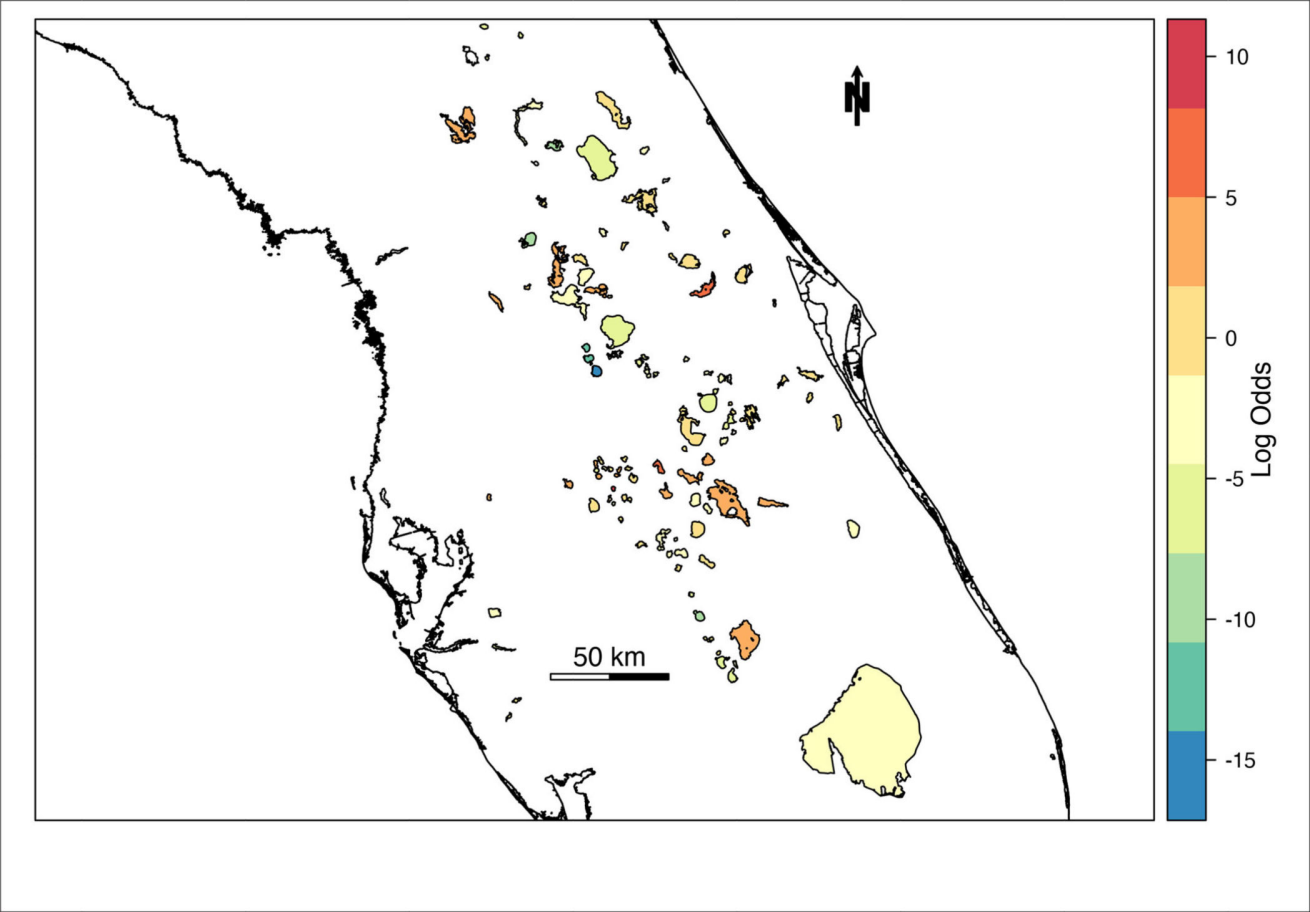
Mean of spatial random effect mapped across lakes in Florida. Lakes with relatively higher log-odds of a high-risk cyanobacteria bloom are indicated with warm colors, while cool colors represent lakes with relatively lower log-odds of high-risk blooms.

The spatial random effect (Figure 4) indicates that there is a significant amount of spatial variation in the mean concentration of cyanoHABs in lakes across Florida. The spatial random effect represents residual error that can be attributed to location and may reflect the influence of an unmeasured or unmeasurable predictor that varies in space. The posterior estimates (mean, standard deviation, 95% CI) for the random hyperparameters are collected in Table 4. The variance of the spatial effect showed a wide posterior distribution (95% CI = 25:56), indicating that the variability in bloom odds attributable to location is high. The posterior mean of the spatial correlation range (the distance at which spatial correlation declines to ~0.1) was 16.8 km with a standard deviation of 3.4 km. This range indicates the approximate distance between lakes within which the odds of an algal bloom can be considered correlated.

### Model Performance

In order to test the performance of the final spatio-temporal model (M4), we used holdout data to determine if M4 correctly estimates the observed data in space and time. Twenty percent (20%) of the data were randomly set aside as a holdout validation dataset *(n =* 2,775 observations). Instead of using an arbitrary cutoff threshold to assign the predicted high-risk bloom value as positive or negative, we calculated a Youden-optimized cutoff point of 0.365. Traditional logistic model evaluation statistics including Area Under Curve (AUC), sensitivity (true positive rate), and specificity (true negative rate) provide insight into the model performance for both holdout dataset scenarios (Table 5). An AUC > 0.5 indicates that the model predicts better than chance alone. The resulting AUC between observations and predictions of the holdout validation dataset was 0.95, sensitivity was 0.88, specificity was 0.88, and accuracy was 0.88.

### Prediction

**FIGURE 5 |:**
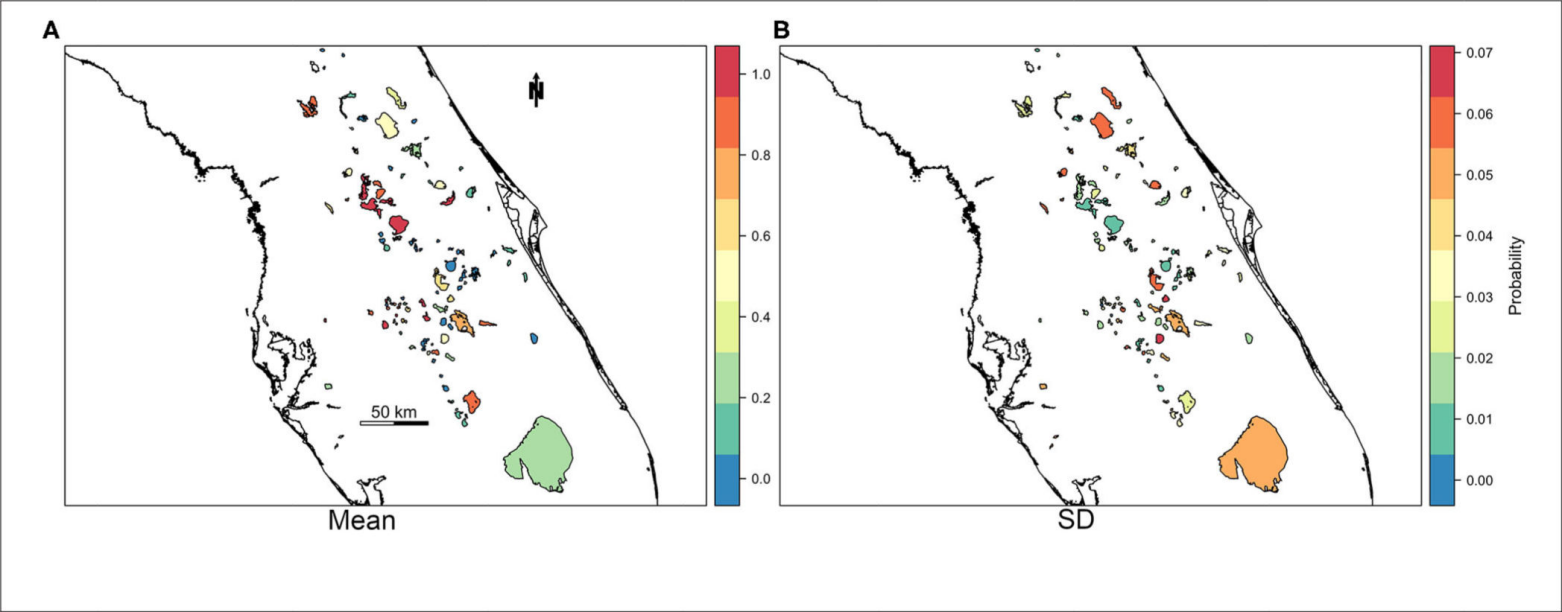
Map of mean **(A)** and standard deviation **(B)** of probability of exceedance of high-risk bloom event for the week of May 27, 2019 through June 2, 2019.

For practical application reasons, we were also interested in assessing the model’s capability to predict or forecast future bloom presence for a week in which the model was untrained. Predictive power of M4 was tested by reserving response and fixed effect data for the most recent week of available data at the time of analysis (May 27th through June 2nd, 2019) as a holdout prediction dataset (*n* = 103 observations). The resulting AUC between observations and predictions of the holdout prediction dataset is 0.89, sensitivity is 0.82, and specificity is 0.82 (Table 5). Our prediction objective was to get a probability of high-risk bloom exceedance, for a given week, using a threshold of 100,000 cyanobacteria cells/mL. As expected, higher exceedance probabilities are detected in lakes with >100 bloom weeks such as Lake Apopka located in central Florida (Figure 5).

## DISCUSSION

Here we present a hierarchical Bayesian spatio-temporal modeling approach in R-INLA to estimate the likelihood of high-risk cyanoHABs in Florida inland waterbodies. Using DIC to evaluate model performance, the full spatio-temporal model (M4, Table 2) was selected as the best model and used to forecast the likelihood of bloom occurrence across Florida lakes for a week outside of the dataset, with AUC 0.93 (Table 5). The spatial random effect (Figure 4) identified residual error attributed to location that may reflect the influence of an unmeasured or unmeasurable predictor that varies across the landscape. The variance of the spatial effect also indicated the variability in cyanoHAB bloom odds for each lake is high. The distance at which spatial correlation declines below a meaningful threshold (0.1) indicates ~17 km as the inter-lake distance within which the odds of cyanoHAB can be considered correlated. Because our model did not include any measures of nutrient input or land use, we believe that it is likely the spatial component of the model is related to land cover and its resultant effect on eutrophication. This landscape effect varies at long spatial scales and is known to have a significant effect on the likelihood of cyanoHABs (Doubek et al., 2015; Marion et al., 2017). Another related possibility is that the spatial correlation represents the distance between watersheds experiencing similar environmental stressors such as nutrient input, making the lakes fed by those watersheds more similar in terms of cyanoHAB risk.

Water surface temperature had a positive effect on cyanoHAB risk, an association that has been observed in many studies to date (Paerl and Huisman, 2008; Wynne et al., 2010; Kosten et al., 2012; Taranu et al., 2012; Beaulieu et al., 2013; Cha et al., 2017). Several mechanisms operate concurrently to the advantage of cyanobacteria at higher water temperatures. The optimal temperature for cyanobacterial growth and photosynthesis is above 20°C, with some species experiencing optimal growth at 30°C or higher (Konopka and Brock, 1978; Lürling et al., 2013; Giannuzzi et al., 2016; Melina Celeste et al., 2017). At high temperatures cyanobacteria have a competitive advantage over green algae, diatoms, and other phytoplankton which favors cyanobacteria dominance. However, some common species of cyanoHAB in the genus *Microcystis* have been found to produce fewer toxins at high temperatures which may mitigate health risks (Runnegar et al., 1983; Rapala et al., 1997; Melina Celeste et al., 2017), although these findings are disputed (Lehman et al., 2008; Davis et al., 2009). Bloom-forming cyanobacteria contain many cylindrical gas vacuoles that impart buoyancy (Walsby, 1977). This buoyancy causes the cells to float to the surface where they are exposed to more light and can outcompete sinking phytoplankton, and there is evidence that increased water temperature improves the ability of cyanobacteria to stay afloat (Kromkamp et al., 1988; Huisman et al., 2004).

Contrary to our expectations, mean lake depth (DMEAN) was a strongly positive correlate of cyanoHAB bloom odds. Our prior belief was that a shallower lake would have a diminished capacity to buffer changes in nutrient input, leading to a heightened risk for HAB blooms. However, in our data this does not seem to be the case. The lakes in our dataset, and Florida lakes in general, are shallow. The average lake in our study had a mean lake depth of only 0.75 m, and the deepest lake had a mean depth of 3.68 m. To contextualize the large positive coefficient of the lake depth effect, we note that an increase in mean depth of 0.75 m, the unit of increase in our model, represents a doubling in depth for the average lake. Satellite penetration in the red spectrum is 2 m or less in oligotrophic waters (Mishra et al., 2005). Given the focus of our model on cyanoHABs at the >100,000 cells/ml threshold, the penetration depth is likely only a few centimeters. It is possible that optically shallow water could cause bottom reflectance or benthic cyanobacteria could cause artifacts. However, Coffer et al. (2020) found the satellite derived phenology of cyanobacteria in Florida followed well-accepted ecological trends and a few lakes that had a peak of biomass in the winter were supported by independent field observations.

Classic models of lake eutrophication posit a strong effect of mixing between the epilimnion and hypolimnion (O’Melia, 1972; Imboden, 1974). Deep lakes are considered to have a greater inertia with respect to changing nutrient conditions; Imboden called this effect a “memory” and found that deep lakes take longer to change from oligotrophy to eutrophy and vice-versa. Shallow lakes do not form the distinctive temperature-delineated layers of a stratified lake and are polymictic: experiencing constant mixing by wind and temperature that leaves the water at a generally homogenous state. This mixing leads to resuspension of nutrients from sediment a higher nutrient load in the water column (Taranu and Gregory-Eaves, 2008; Liu et al., 2012). The lakes in our study are not deep enough to stratify and should be considered polymictic. Because all of our lakes are shallow, nutrients cannot sequester within the hypolimnion and sediment and the association between depth and oligotrophy found in stratified lakes does not apply.

In a study of cyanobacteria growth in shallow lakes (<6 m), Kosten et al. found similar results to our study, with water temperature predicting a higher proportion of cyanobacteria among the phytoplankton community (Kosten et al., 2012). A study of the National Lake Assessment dataset, containing 1,147 lakes and spanning the continental U.S., found that when lakes were divided into shallow (<6 m) or deep, their models were less able to predict cyanobacterial concentrations in the shallow lakes (Beaulieu et al., 2013). Associations of HAB volume with nitrogen content and water temperature were consistent across all lakes, but there was a decrease of two-thirds in variation explained when lakes were shallow. A national-scale study in the United Kingdom that investigated both shallow and deep lakes suggested that water residence time, a correlate of depth, was a positive predictor of HAB biovolume, especially in lakes with significant opacity (Carvalho et al., 2011). An in-depth study of a shallow (mean depth 1.2 m), warm, polymictic lake in the Mediterranean that was substantially similar to the lakes in our study found that water residence time was a strong predictor of harmful algae concentration (Romo et al., 2013). We propose that in our study, mean lake depth is acting as a correlate of water residence time, explaining its positive association with HAB blooms.

Air temperature had a negative effect on cyanoHAB risk, a result that seems counterintuitive when considered alongside the positive effect of water surface temperature. Our model suggests that cyanoHAB risk is heightened when air temperature is lower relative to surface water temperature, a condition that can occur in shallow lakes during the transition from summer to fall, or when lake temperature is artificially increased by impervious surface runoff or industrial input (Sabouri et al., 2013). In our dataset, the peak mean water temperature was significantly higher than the peak mean air temperature observed (36.5 and 30.3°C, respectively). The higher specific heat of water is responsible for this effect, with lakes serving as a heat sink that can persist after air temperature has cooled.

The predictive accuracy of our model on a holdout dataset was high, with ~82% of held-out observations for the week of May 27, 2019 predicted correctly. Accuracy was also good in our validation dataset, with 88% of observations correctly classified. The accuracy of our simple model is encouraging but has a few caveats. This model is not expected to remain accurate for more than 2 weeks into the future, because wide spatial coverage meteorological estimates become less reliable beyond that time scale. Additionally, the autocorrelation parameter, or *α*, was 0.9, indicating that the change in the temporal component of risk of a cyanoHAB is strongly related to the past week’s bloom conditions. While this temporal component was not as significant a contribution to model fit as the spatial component that considered lake location (Table 2), we caution that interpretation of our model’s accuracy should consider that due to system inertia, a fairly accurate prediction of near-future bloom conditions can be made by simply extrapolating that current conditions will continue unchanged.

Although we have developed a reliable model for forecasting cyanoHAB odds for lakes in central Florida lakes for a week of interest outside of the modeled dataset, there are several limitations of this study. While remote sensing provides a continuous cyanoHAB data source, the relatively coarse 300 m sensor resolution, presence of cloud cover, and occasional missing data due to waterbody misclassification, limits our predictive ability in smaller inland waterbodies. Additional satellite limitations are reviewed in Coffer et al. (2020) and Clark et al. (2017). Future work could assess the applicability of higher resolution sensors such as the European Commission’s Copernicus Sentinel-2. Our covariates were not all available at the same spatial scale and had to be resampled to the same 300 m resolution as our lake imagery. As a result, lake areas with highly irregular or elongated narrow reaches may be underrepresented vs. lake areas with broader widths. It is possible that CyanoHAB was present in a specific part of the lake, but satellite data didn’t capture it because of land proximity. This study incorporated several environmental covariates known to be associated with cyanoHAB occurrence; however, our results showed a strong spatially varying effect, the cause of which is undetermined in the present analysis. Future studies could explore additional environmental determinants such as nutrient loadings. Nutrients certainly play a role related to cyanoHABs and nutrient eutrophication is a “wicked” problem as defined by Thornton et al. (2013), meaning that the issue is convoluted and precludes a simple solution. However, modeling nutrient transport and availability is complex and system specific, with model validation data lacking for most systems. This complexity, specificity and lack of data limits the ability to scale such models across spatial and temporal scales as described in Soranno et al. (2015). Therefore, we limited the model to data that would be readily available and most relevant to cyanoHAB biomass.

In the present study we applied R-INLA for the purpose of mapping/predicting exceedance probability of high-risk cyanoHABs at the level of a whole lake. Future work will apply the spatial-temporal modeling approach at the sub-lake level, particularly in large systems with greater geographic bloom variability such as Lake Okeechobee in South Florida.

## Supplementary Material

Supplement1

## Figures and Tables

**TABLE 1 |:** Summary information for environmental datasets used for covariate selection.

Source	Variable name	Abbreviation	Units	Resolution

PRISM (Daly et al., 1997)	Air temperature	ATEMP	Degrees celsius (°C)	4 km; daily
	Precipitation	PRECIP	Millimeters rainfall (mm)	13.8 km; hourly
Landsat Analysis Ready Data (ARD) (Cook et al., 2014)	Surface water temperature	WTEMP	Degrees celsius (°C)	30 m; 16 and 8-day
lakeMorpho R-package (Hollister and Stachelek, 2017)	Surface area	Area	Square meters (m^2^)	Static
	Mean lake depth	dMean	Meters (m)	Static

**TABLE 2 |:** Models considered with corresponding performance information.

Model	Model description	DIC	Computation time (s)

M1	Non-hierarchical model	21,381	∼7
M2	Temporal component only	21,294	∼10
M3	Spatial component only	7,758	∼45
M4	Spatial + temporal model	7,601	∼53

**TABLE 3 |:** Posterior estimates (mean, St. Dev., quantiles) for fixed effects.

Variable	Mean posterior	St. Dev.	0.025	0.5	0.975

ATEMP	−0.23	0.07	−0.37	−0.23	−0.08
WTEMP	0.17	0.05	0.08	0.17	0.26
PRECIP	−0.01	0.04	−0.09	−0.01	0.07
AREA	−0.11	0.66	−1.40	−0.11	1.19
DMEAN	2.70	0.52	1.68	2.70	3.72

**TABLE 4 |:** Posterior estimates (mean, St. Dev., quantiles) for random effects.

Parameter (random effect)	Posterior mean	St. Dev.	0.025	0.5	0.975

Temporal variance	0.19	0.09	0.05	0.15	0.62
Spatial variance	38.51	7.85	25.63	37.61	56.35
Spatial correlation range *ρ*	16.76	3.44	10.72	16.54	24.14
AR (1) parameter *α*	0.90	0.09	0.68	0.92	0.99

**TABLE 5 |:** Results of the statistical evaluation metrics obtained using a cutoff point of 0.365.

Metric	Validation dataset	Prediction dataset

AUC	0.95	0.89
Sensitivity	0.88	0.82
Specificity	0.88	0.82
Accuracy	0.88	0.82

## Data Availability

The datasets presented in this study can be found in online repositories. The names of the repository/repositories and accession number(s) can be found at: US EPA Environmental Dataset Gateway, https://edg.epa.gov/metadata/catalog/main/home.page. DOI: 10.23719/1518919.
