# Supplementary material for: Spatio-Temporal Modeling for Forecasting High-Risk Freshwater Cyanobacterial Harmful Algal Blooms in Florida
Source: Front Environ Sci. Author manuscript; Available in PMC 2021 Nov 2. (PMC7751622)
Supplement: Supplement1 [file NIHMS1645363-supplement-Supplement1.zip › Data_Sheet_1_Spatio-Temporal Modeling for Forecasting High-Risk Freshwater Cyanobacterial Harmful Algal Blooms in Florida.DOCX]

***Supplementary Material***

1. **Supplementary Tables**

| **Table S1.** Descriptive statistics for variables considered in the final model. | | |
| --- | --- | --- |
| Variable (units) | Mean ± SD | Range (min : max) |
| ATEMP (°C) | 23.22 ± 4.79 | 5.14 : 30.33 |
| WTEMP (°C) | 21.78 ± 6.23 | 0.35 : 36.56 |
| PRECIP (mm) | 4.15 ± 5.26 | 0 : 46.01 |
| AREA (km^2^) | 35.16 ± 165.84 | 1.56 : 1674.90 |
| DMEAN (m) | 0.79 ± 0.75 | 0 : 3.68 |

1. **Statistical Appendix**

The definitions, explanations, and formulas presented here are intended to aid in interpreting the results of our paper and provide an interested non-statistical reader the background information necessary to contextualize our results. For a rigorous mathematical discussion on these concepts the authors encourage the consultation of a current college-level textbook on statistical modeling.

## Bayesian model

A Bayesian model is a statistical method in which the researcher’s previous knowledge about the possible distribution of the data (referred to as a **prior**) interacts with the distribution of the observed data (referred to as the **likelihood**) to produce a statistical statement about the observations (referred to as a **posterior**). This kind of model is named after an 18th century mathematician named Thomas Bayes, whose eponymous theorem (Formula S1) forms the basis of the model. Stated in general terms, Bayes’ Theorem says that the probability of event A, given that event B has already occurred, is equal to the probability of event B given that event A has already occurred multiplied by the probability of event A occurring, divided by the probability of event B occurring. This concept can be extended, using complex computational methods that are best done by a computer, to estimate parameters of statistical models. In Formula S1, *θ* refers to the statistical parameters of the model, *x* refers to the data, and *P(x)* refers to the probability of *x*. *P(x|θ),* or the probability of observing the data given the model parameters, is referred to as the *likelihood function* of the model. Bayesian modeling is the computation of estimates for the parameters of interest (which may include predictor variable coefficients, means, or any other statistic) from priors and data.

$P(\theta|x) = \frac{P(x|\theta) \times P(\theta)}{P(x)}$

Formula S1. Bayes’ Theorem.

## Generalized linear model (GLM)

A linear model is a kind of regression that estimates the values of a continuous variable *y* given predictor covariates *x*, by using the method of least squares (Miller 2006) to fit the values of *y* to a line whose slope is *ꞵ*, which is referred to as the regression coefficient. In a simple linear model, also known as a linear least squares regression, it can be said that for a 1-unit increase in *x*, there is a corresponding *ꞵ*-units increase in *y*, where *y* starts off at an intercept *a* when *x* is equal to 0. (Formula S2).

$y_{i}=a +\beta x_{i}+ \varepsilon_{i}$

Formula S2. Simple linear model for observation *i* with residual error represented by *ε*.

The simple linear model can be extended to encompass multiple covariates and methods for categorical variables such as ANOVA and its derivatives. This extension is known as a general linear model (Formula S3).

$$y_{i}=a+\sum_{j=0}^{n_{\beta}} \beta_{j}x_{ij}+ \varepsilon_{i}$$

Formula S3. General linear model for observation *i* with *j* covariates and residual error represented by *ε*. This case is also known as a multiple linear regression.

The general linear model can be further extended to encompass relationships between *y* and *x* that are nonlinear, such as logistic or Poisson regression. These use a link function, commonly referred to as *g*, to describe the relationship between the (sometimes nonlinear) distribution of the response variable *y*, and the linear predictor *η = βx*. The link function *g* describes how the *mean expected value of* *y*, referred to as *μ*, depends on the linear predictor, and can be any of a large group of nonlinear model types (Formula S4).

$${g(\mu}_{i})=\eta=a+\sum_{j=0}^{n_{\beta}} \beta_{j}x_{ij}+\varepsilon_{i}$$

Formula S4. Formula for a generalized linear model. The link function *g* describes the relationship between the linear predictor *η* and the mean expected value of *y*, called *µ*.

## INLA

Integrated Nested Laplace Approximations (INLA) is a way to estimate the parameters of difficult-to-compute distributions. It is based on the Taylor expansion method that is used in calculus to approximate functions at values of interest. The details are complex and involve dozens of equations (Rue et al. 2009). In short, many Bayesian models that are mathematically difficult to solve by traditional techniques or by previous computer-assisted techniques may be solved by INLA because it allows a very close approximation of a function, which has much simpler mathematical properties, to stand in for that function in the computation of parameters of the model. In our paper, we used a package called R-INLA ([www.r-inla.org](http://www.r-inla.org)) provided in the statistical programming language R to solve our models.

## SPDE

## Stochastic Partial Differential Equations (SPDE) are a similar approach to INLA, in that they are a mathematical way to simplify a type of mathematical calculation that was previously difficult or impossible for even a computer to solve (Lindgren et al. 2011). By using SPDE, many kinds of models, including spatial models with many parameters that are computationally complex, can be thought of as a particular collection of normally distributed linear equations, or a Gaussian Markov Random Field. These equations are amenable to computation by the INLA approach, and therefore render previously intractable spatial and spatiotemporal models solvable by a computer.

## Delaunay Triangulation

## Delaunay triangulation is a method of dividing a plane containing points into triangles such that the circumcircles of each triangle pass through only those points. This method maximizes the minimum angle of all the triangles in the plane and tends to result in a mesh of somewhat equally proportioned triangles that minimizes small slivers. It is useful in spatial modeling because of it creates a subset of regions on which to calculate the values of the model. The Delaunay triangulation mesh used in our model is given in Figure S1. For our model, we used a mesh that was denser (*i.e.* contained more triangles) near lakes, because we were only interested in plotting modeled spatial coefficients in lakes. The values of the spatial effect over land are useful only for computing the relationships between the distance at which the spatial effects are correlated among lakes, and so the mesh can be coarser over these areas to dramatically reduce computing demand.


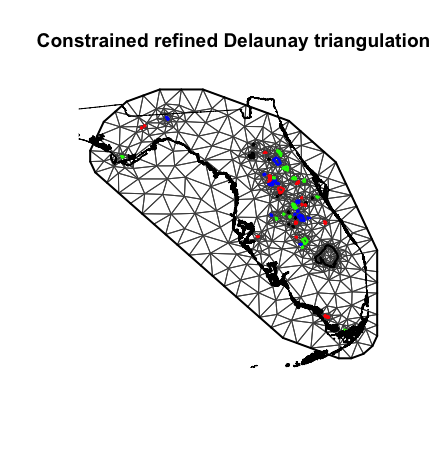


Figure S1. Delaunay triangulation mesh on which the values of the spatial model were defined. Regions in thick black and multicolored outline represent lakes (the colors were added to help differentiate lakes that are close together). The silhouette of Florida has been superimposed for reference.

## Autoregressive Model, Order 1 (AR1)

An autoregressive model estimates a result that is dependent on its previous states. For this reason, it is often used in forecasting or prediction when one of the covariates is time. One of the decisions that must be made when using an autoregressive model is how many previous states to take into account when estimating the current step; in an AR(1) model, only the immediately previous state is used. The equation for an AR(*p*) model, where *p* is the model order, is as follows (Formula S5):

$$X_{t}=c+\sum_{i=1}^{p} \varphi_{i}X_{t-i}+\varepsilon_{t}$$

Formula S5. Autoregressive model of order *p*, where *φ* are the parameters of the model, *t* refers to the current time step, *c* is a constant, and *ε* is an error term.

## Akaike’s Information Criterion (AIC)

The AIC (Formula S6) is a measure of model fit that is derived from the log-likelihood value (itself a measure of model fit, please see Penn State, n.d. for information on how it is calculated), penalized by the number of parameters estimated by the model (*k*). It is a way to correct for the bias towards complex models in model fitting. When comparing candidate models, a lower value is considered a better fit.

$$AIC= -2(log likelihood)+2k$$

Formula S6. Akaike’s Information Criterion.

## Deviance Information Criterion (DIC)

The DIC (Formula S7) is a generalization of the AIC often used for Bayesian model comparison (Spiegelhalter et al. 2002). To calculate the DIC, one must estimate the number of parameters in the model because in complex Bayesian models this value can be unclear. This calculation can be complicated and is usually done by a computer; DIC is rarely calculated by hand.

$$DIC = D(\theta)+2p_{D}$$

where

$$D(\theta) = -2log(p(y|\theta))$$

and

$$p_{D}=\frac{1}{2}var(D(\theta))$$

Formula S7. Deviance Information Criterion, where *θ* are the unknown parameters of the model, *y* are the data, *p(y|θ)* is the likelihood function for the model (See the definition for **Bayesian model** above). *Var(D(θ))* is the variance of the estimated log-likelihood.

## True Positive Rate (TPR); Sensitivity

## The True Positive Rate, also referred to as the sensitivity of a model, is the proportion of positive predictions in a binary classification model that match actual positives (Formula Sn).

$$TPR= \frac{Predicted positive outcomes}{Actual positive outcomes}$$

Formula S8. True positive rate.

## True Negative Rate (TNR); Specificity

The True Negative Rate, also referred to as the specificity of a model, is the proportion of negative predictions in a binary classification model that match actual negatives (Formula Sn).

$$TNR= \frac{Predicted negative outcomes}{Actual negative outcomes}$$

Formula S9. True negative rate.

## Accuracy

The accuracy of a binary classification model is the proportion of predictions that are correct (Formula Sn).

$$Accuracy= \frac{True Positives+True Negatives}{Number of Observations}$$

Formula S10. Accuracy.

## Receiver Operating Characteristic Curve (ROC)

The Receiver Operating Characteristic (ROC) curve is a visualization of the trade-off between sensitivity and specificity when setting a cut-off point in a binomial classification model. The output of a binomial model is a number between 0 and 1, and in order to make predictions based on the model a cut-off point must be chosen above which a prediction is considered positive, and below which it is considered negative. Intuitively, one might set this value to 0.5, but that does not necessarily deliver the best accuracy. In order to deliver the most accurate predictions based on the model, a ROC curve can be drawn (Figure S2).


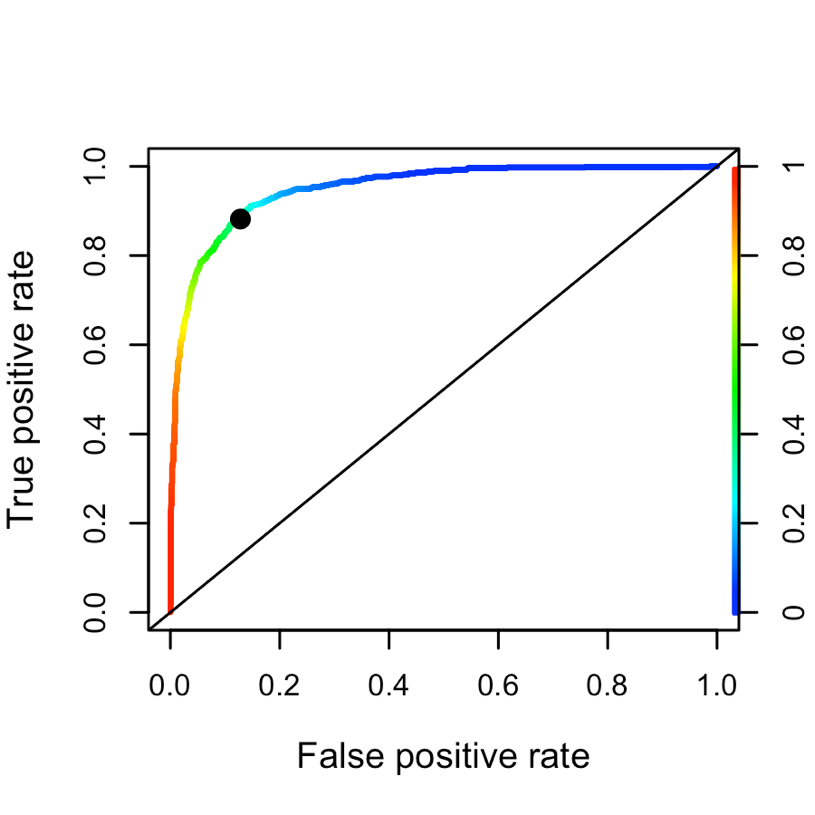


Figure S2. ROC curve for our final model. The colors on the right vertical axis represent the values of the cutoff point, from 0 to 1.

This curve has the true positive rate on the Y-axis, and the false positive rate (1-TNR) on the X-axis. The line represents all possible values of the cut-off point for the model, and the black dot is the optimal point at which TPR is maximized and TNR is minimized. This cutoff point results in the best accuracy value. We note that overall accuracy may not be the criterion for which a modeler may wish to optimize: consider a test for an acute, deadly disease. In that case, more false positives may be acceptable in order to ensure that all true positives are detected. An ROC curve is only one method to help optimize a binomial model.

## Area Under Curve (AUC)

The Area Under Curve value is the integral of the ROC curve. It measures the overall quality of the model’s predictions irrespective of which cutoff rate is chosen. Because of this, the AUC value can be used to rank binomial models.

1. **References**

Cook, M., Schott, J., Mandel, J., & Raqueno, N. (2014). Development of an operational calibration methodology for the Landsat thermal data archive and initial testing of the atmospheric compensation component of a Land Surface Temperature (LST) product from the archive. Remote Sensing, 6(11), 11244-11266.

Daly, C., Taylor, G. H., & Gibson, W. P. (1997). The PRISM approach to mapping precipitation and temperature. In *Proc., 10th AMS Conf. on Applied Climatology* (pp. 20-23).

Hollister, J., & Stachelek, J. (2017). *lakemorpho*: Calculating lake morphometry metrics in R. *F1000Research*, *6*, 1718. <https://doi.org/10.12688/f1000research.12512.1>.

Lindgren, F., Rue, H., & Lindström, J. (2011). An explicit link between Gaussian fields and Gaussian Markov random fields: the stochastic partial differential equation approach. Journal of the Royal Statistical Society: Series B (Statistical Methodology), 73(4), 423-498.

Miller, S. J. (2006). The method of least squares. *Mathematics Department Brown University*, *8*, 1-7.

Penn State. (n.d.). STAT 504 – Analysis of Discrete Data. Section 1.4: Likelihood and Log-Likelihood (MOOC). Penn State Eberly College of Science. https://online.stat.psu.edu/stat504/node/27/.

Rue, H., Martino, S., & Chopin, N. (2009). Approximate Bayesian inference for latent Gaussian models by using integrated nested Laplace approximations. Journal of the royal statistical society: Series b (statistical methodology), 71(2), 319-392.

Spiegelhalter, D.J., Best, N.G., Carlin, B.P. and Van Der Linde, A. (2002), Bayesian measures of model complexity and fit. Journal of the Royal Statistical Society: Series B (Statistical Methodology), 64: 583-639. doi:[10.1111/1467-9868.00353](https://doi.org/10.1111/1467-9868.00353)
